# CERC: an interactive content extraction, recognition, and construction tool for clinical and biomedical text

**DOI:** 10.1186/s12911-020-01330-8

**Published:** 2020-12-15

**Authors:** Eva K. Lee, Karan Uppal

**Affiliations:** 1 Center for Operations Research in Medicine and HealthCare, School of Industrial and Systems Engineering, School of Biological Sciences, Georgia Institute of Technology, Atlanta, USA; 2 School of Medicine, Emory University, Atlanta, GA USA

**Keywords:** Automatic summarization, Content extraction and recognition, Extractive summarization, Indicative summarization, Sentence extraction and ranking, Extracting salient information, Machine learning, Multiple indicators, Multi indicator text summarization algorithm, Automatic translation, Clinical decision support

## Abstract

**Background:**

Automated summarization of scientific literature and patient records is essential for enhancing clinical decision-making and facilitating precision medicine. Most existing summarization methods are based on single indicators of relevance, offer limited capabilities for information visualization, and do not account for user specific interests. In this work, we develop an interactive content extraction, recognition, and construction system (CERC) that combines machine learning and visualization techniques with domain knowledge for highlighting and extracting salient information from clinical and biomedical text.

**Methods:**

A novel sentence-ranking framework multi indicator text summarization, MINTS, is developed for extractive summarization. MINTS uses random forests and multiple indicators of importance for relevance evaluation and ranking of sentences. Indicative summarization is performed using weighted term frequency-inverse document frequency scores of over-represented domain-specific terms. A controlled vocabulary dictionary generated using MeSH, SNOMED-CT, and PubTator is used for determining relevant terms. 35 full-text CRAFT articles were used as the training set. The performance of the MINTS algorithm is evaluated on a test set consisting of the remaining 32 full-text CRAFT articles and 30 clinical case reports using the ROUGE toolkit.

**Results:**

The random forests model classified sentences as “good” or “bad” with 87.5% accuracy on the test set. Summarization results from the MINTS algorithm achieved higher ROUGE-1, ROUGE-2, and ROUGE-SU4 scores when compared to methods based on single indicators such as term frequency distribution, position, eigenvector centrality (LexRank), and random selection, *p* < 0.01. The automatic language translator and the customizable information extraction and pre-processing pipeline for EHR demonstrate that CERC can readily be incorporated within clinical decision support systems to improve quality of care and assist in data-driven and evidence-based informed decision making for direct patient care.

**Conclusions:**

We have developed a web-based summarization and visualization tool, CERC (https://newton.isye.gatech.edu/CERC1/), for extracting salient information from clinical and biomedical text. The system ranks sentences by relevance and includes features that can facilitate early detection of medical risks in a clinical setting. The interactive interface allows users to filter content and edit/save summaries. The evaluation results on two test corpuses show that the newly developed MINTS algorithm outperforms methods based on single characteristics of importance.

## Background

Implementation of electronic health record systems (EHRs) across healthcare institutions and growing information in biomedical databases provides a unique opportunity to enhance clinical decision-making by linking patient-specific information with scientific literature to support clinicians’ needs [1]. However, this is a challenging task due to the rapid and exponential growth of data and information sources. The burden of “information overload” demand that intelligent informatics tools and algorithms be advanced to automate the processing of large amounts of text to uncover knowledge [2, 3]. According to a recent review, almost half of the questions related to patient care raised by clinicians are not pursued due to limited amount of time at point of care and doubts about availability of information [4]. Although most scientific articles include abstracts, recent studies have shown the advantages of using full-text for summarization since not all relevant information can be reported in abstracts [5]. Moreover, different readers may find different pieces of information in the text useful [6].

The problem of information overload is also associated with EHRs since the amount of stored clinical information per patient could be excessive, particularly for patients suffering from chronic illness and multi-morbidities [2, 7, 8]. A cognitive study of the thought process of eight physicians during the EHR review process showed that majority of their time is spent reviewing the “Notes” section to identify problems, medical history, medications, etc. [7]. Text mining and natural language processing techniques have the potential to enhance clinical-decision making and improve the quality of healthcare [9–15]. For instance, studies have shown their utilization can facilitate detection of adverse drug events and comorbidities in EHRs [11, 12]. It has also been shown that high-information clinical findings appear in the medical records of patients before the high-risk diagnosis is determined [13]. Furthermore, automated summarization of patient information to extract salient information can improve decision-making and reduce the risk of information overload [7, 14, 64].

In this work, we develop machine learning based automated text summarization techniques to address the challenges of “salient detection” and “information overload” in healthcare and biomedical domains [2, 16]. Automated summarization aims to extract important information from the original text and present it in a condensed form [16–18]. Summarization methods can be classified as extractive versus abstractive [17]. Extractive summarization involves extracting important sentences from the input text according to a scoring or ranking criteria, while abstractive methods use natural language processing techniques to construct new sentences [5, 18]. The two categories can be further classified as indicative versus informative where indicative summaries only provide an overview of the underlying information, while informative summaries provide enough details to replace the original text [16]. Various extractive summarization methods have been developed over the last decade [19–23]. These methods utilize a variety of sentence ranking strategies such as intermediate topic representation, graph-based methods based on Google PageRank algorithm and UMLS semantic relations in UMLS (http://www.nlm.nih.gov/research/umls/), MeSH terms, sentence position, and semantic relations of biomedical concepts [23]. For example, Bhattacharya et al. demonstrated that usage of MeSH terms improves summarization results, Fiszman et al. used semantic relationships for summarization of Medline citations, Reeve et al. used the concept frequency for summarization, Jonnalagadda et al. used UMLS concepts and TextRank algorithm for extracting sentences related to a particular topic from Medline abstracts, and Mishra et al. used clinically relevant sentences from UpToDate [17–25]. Most existing extractive summarization methods utilize single indicators of relevance for sentence ranking that might not be relevant for all types of clinical and biomedical use cases. Human knowledge can enhance the effectiveness of data mining and exploration process. Users can interact with summarization system via visualization tools that provide insight into the underlying information [26–28].

In this paper, we present CERC, a content extraction, recognition, and construction visualization tool that uses a multi-stage sentence evaluation and ranking framework for extracting salient information from the input text. A random forests classifier is used in stage one for evaluating worthiness (“important” versus “not important” for summarization) of each sentence in the input text. In stage two, a rank aggregation scheme based on multiple indicators is used for identifying the best set of sentences to be included in the final summary. The performance of CERC was evaluated against existing summarization techniques using a subset of articles from the Colorado Richly Annotated Full Text (CRAFT) corpus and a corpus of full-text clinical case reports obtained from Medline [29]. Indicative summarization is performed using an interactive topic cloud based on over-represented biomedical terms in the input text. The topic cloud provides a visual overview of the content in the input text and allows interactive filtering of the sentence extraction results based on users’ interests. A keyword-based filtering allows users to generate a summary based on the top-ranked sentences and edit and save the selected summary for future review or additional processing such as language translation [41, 42, 64]. Finally, related articles in PubMed are presented based on the topic cloud to incorporate external knowledge.

The main objectives of this research are: (1) development of extractive and indicative summarization algorithms to address information challenges related to precision medicine; (2) development of a web-based interactive summarization tool that accounts for user specific interests and can facilitate clinicians in summarizing clinical/biomedical text by highlighting key information both at the level of individual terms and sentences. We also demonstrate the integration of CERC within clinical decision support systems for direct patient care.

## Materials and methods

**Fig. 1:**
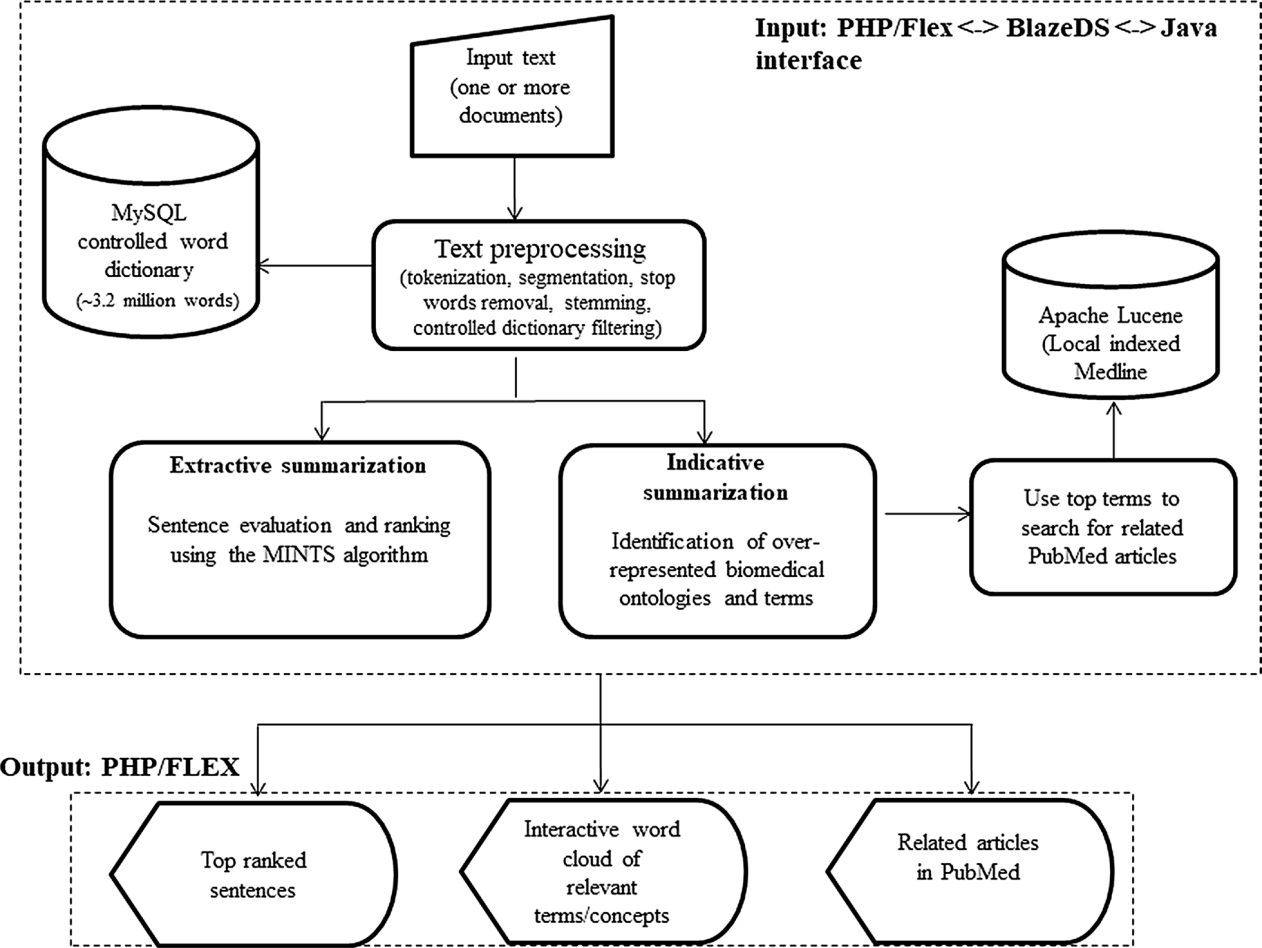
CERC system diagram. This shows the overall design of CERC. Input text is first preprocessed via segmentation, tokenization, stemming, and controlled dictionary filtering. Extractive summarization is performed using a new algorithm, the MINTS algorithm, a multi-stage algorithm for sentence extraction and ranking. A word-cloud based visualization method is used to represent term/concept distribution

Figure 1 shows the overall design of CERC. Input text is first preprocessed via segmentation, tokenization, stemming, and controlled dictionary filtering. Extractive summarization is performed using a new algorithm, MINTS, a multi-stage algorithm for sentence extraction and ranking. A word-cloud based visualization method is used to represent term/concept distribution. Below, we detail each of the components in CERC.

### Preprocessing: segmentation, tokenization, and stemming

Segmentation of input text into individual sentences is performed using the LingPipe tool kit (http://alias-i.com/lingpipe/). The input text is segmented into word tokens using regular expression rules. Porter stemmer algorithm is used to reduce all inflected forms of a word to the same text string, eg: {densities, density} -> densiti [30].

### Indexed database of Medline abstracts [44]

Apache Lucene [31] is used to generate an indexed database of Medline abstracts published between 1975 and 2015. Lucene, a text search engine written in Java, facilitates efficient querying and document retrieval.

### Dictionary of controlled vocabulary and stop words [44]

A controlled dictionary of 3.2 million words was generated using MeSH terms, SNOMED-CT, and PubTator, which includes terms related to genes, proteins, genetic variants, taxonomy, diseases/disorders, and chemicals from biomedical literature [32–34]. In addition to the 121 stop words used by PubMed (https://www.ncbi.nlm.nih.gov/books/NBK3827/table/pubmedhelp.T.stopwords/), any word in the input text that is not present in the controlled dictionary is considered a stop word.

### Extractive summarization: MINTS: a multi-stage algorithm for sentence extraction and ranking

**Fig. 2:**
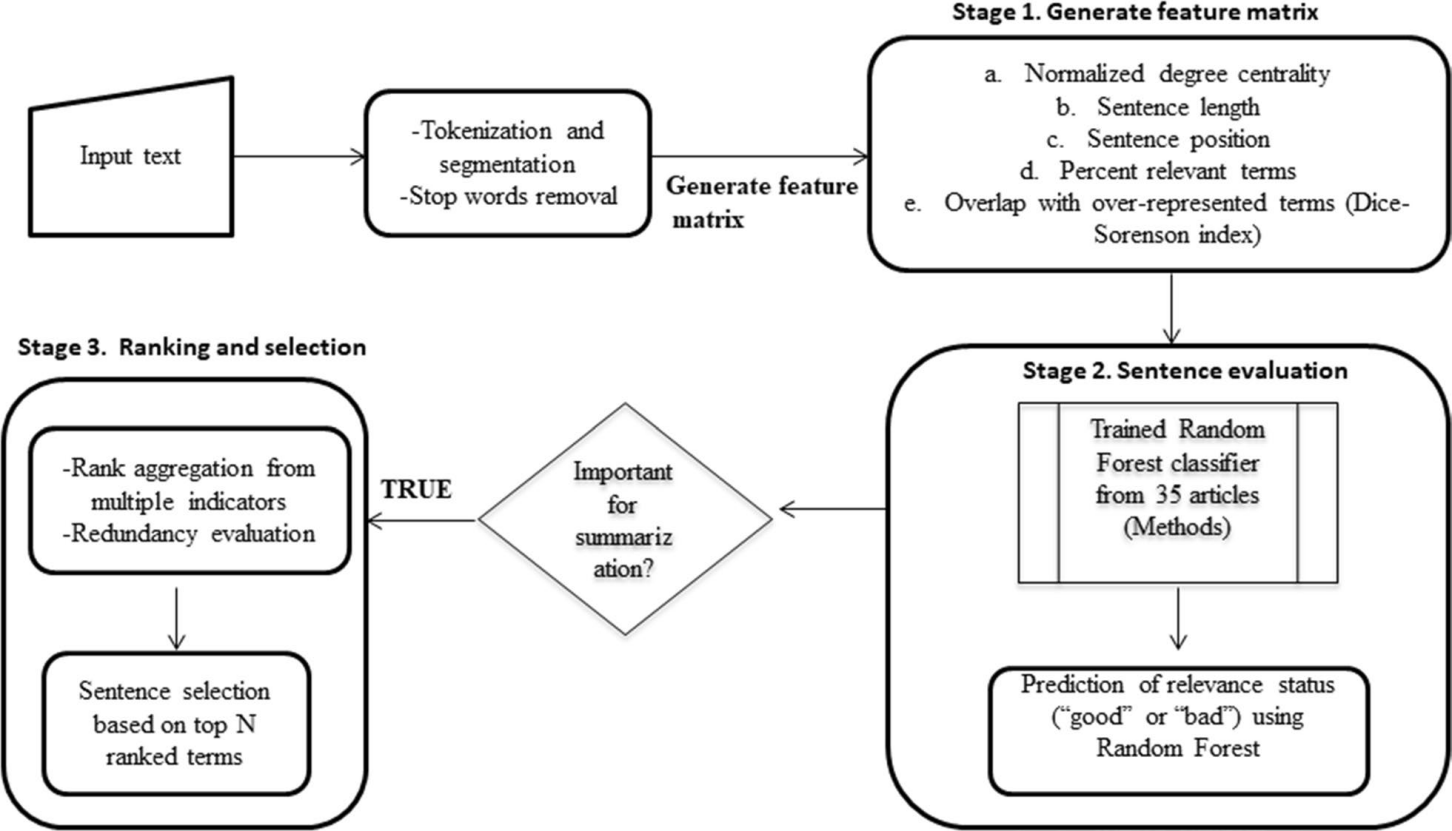
MINTS workflow for extracting salient sentences. MINTS uses a multi-stage framework that combines supervised learning techniques, individual characteristics of sentences (position, length, relevant terms) and network level characteristics (degree centrality) for extracting salient sentences

Machine learning techniques such as decision trees, hidden Markov Model, and Naïve Bayes classifier, etc. have been implemented for sentence extraction [17, 18]. Some of the common importance indicators utilized by previous machine learning methods include sentence length, position, term frequency–inverse document frequency (TF-IDF), and parts of speech [18]. In this work, we developed a three-stage procedure to extract relevant sentences using a random forests classifier and various indicators of relevance such as: sentence length, position in the input text, number and percentage of clinical/biomedical terms, normalized degree centrality, and overlap with global term frequency distribution determined using the Sørensen–Dice-coefficient/index (DS) as similarity metric [20, 35, 45, 46]. We called this new algorithm the multi indicator text summarization algorithm, MINTS (Fig. 2).

In stage one, a sentence-feature matrix is generated where each row corresponds to an individual sentence and the columns represent the indicators of relevance. The number of domain-specific terms is determined using the controlled dictionary. A TF-IDF based cosine similarity matrix is used to determine the degree centrality of each sentence, which is normalized by the total number of sentences in the input text [18]. According to Luhn’s theory, the most frequent terms/concepts are the most important ones and can be used to determine the significance of individual sentences [5, 35]. The overlap between the term frequency distribution of the current sentence and the global frequency distribution is determined using Sørensen-Dice-coefficient, Eq. (1), as similarity metric, which has been previously shown to outperform other similarity function metrics for determining the overlap between a candidate summary and the source text [21].

1 $${\text{DS}} { }\left( {\text{s}} \right) = { }2{*}\frac{{\left| {{\text{A }} \cap {\text{B}}} \right|}}{{\left| {\text{A}} \right| + \left| {\text{B}} \right|}}$$

where s = index of current sentence, $\left| A \right|$ = number of relevant terms/concepts in the frequency distribution model of the entire document, $\left| B \right|$= number of relevant terms/concepts in the frequency distribution model of sentence s, $|A \cap B|$= number of overlapping terms/concepts between the global frequency distribution model and the distribution model of sentence s.

In stage two, a random forests classifier is used to predict the “worthiness” of a sentence. Random forests is a non-parametric supervised classification technique that uses an ensemble of decision trees for learning a model [36]. Each tree in the forest is generated using a random set of variables (relevance indicators) and by sampling a random set of training samples (bagging). The trees are grown until the leaves/terminal nodes contain samples belonging to the same class. After the forest is constructed, every tree casts a vote for the class assignment of the new sample. The class of the new sample is determined using the majority vote. The randomForest package in R is used in CERC.

We used CRAFT to evaluate the performance quality of the system. These 67 articles have each been annotated with nine biomedical concepts and syntactics. The biomedical concepts include cell, protein, and sequence ontologies, the entries of the Entrez Gene database, and the 3 gene subontologies (biological processes, cellular components and molecular functions), the Chemical Entities of Biological Interest ontology, and the NCBI Taxonomy. Each sentence has been marked up to sentence segmentation, tokenization, part-of-speech tagging, and coreference. The syntactic parses, manually curated for each sentence, are represented in Penn Treebank format.

Thirty-five full-text articles from the CRAFT corpus were used to build the random forests model. The remaining 32 articles were used during the blind evaluation stage as described later. The two sets will be termed as CRAFTtrain and CRAFTtest, respectively. All articles were preprocessed to remove stop words. Since it is challenging to manually annotate each sentence in full-text articles, a data-driven approach was used to annotate the 9,779 sentences in the CRAFTtrain corpus. Each sentence was annotated as “good” or “bad” using the DS index (Eq. 1) which is based on the amount of overlapping terms between the sentence and the article abstract. The maximum DS index values per article ranged from 0.046 to 0.358 in CRAFTtrain corpus with a median value 0.038 and 25^th^ percentile value of 0.01 across all sentences Sentences with a DS index less than 0.01 were annotated as “bad” or not important for summary. This resulted in 7,498 out of 9,779 sentences being annotated as “good” for summarization. A random forests model was trained using 60% of the sentences (N = 5867; N_good_ = 4401; N_bad_ = 1466), and its performance being evaluated using the remaining 40% of the sentences (N = 3912; N_good_ = 3,097; N_bad_ = 815). The trained classifier is used to evaluate the importance of every sentence in the new text. And only those sentences that are predicted as “good” are used in the ranking stage. This facilitates document compression/data reduction.

The last stage involves selection of “good” sentences for generating summaries based on aggregated ranking and redundancy evaluation. The scores based on *m* indicators for every sentence are converted to ranks, *Rim* = [*1…N*], where *i* is the sentence index, *m* is the indicator of importance (e.g. degree centrality, position, etc.), and *N* is the number of sentences. Each sentence is assigned an aggregated rank, calculated as the average of rankings from different indicators. The top ranked sentences are used for summaries after evaluating the cosine similarity (a threshold of 0.4 is used based on empirical evaluation) between the previously selected sentences in the summary set and the incoming sentence to reduce redundancy [18]. A normalized score ranging between 0 (least important) to 1 (most important) is assigned to each sentence. Users can input the maximum number of sentences to be selected. The default is set at 5 sentences.

For comparison purposes, topic-based and graph-based extraction summarization techniques were also included during the evaluation process:

i topicDist: This method evaluates the relevance of a sentence term/concept frequency based on the overlap with the most frequent terms/concepts in the entire text [5, 21, 35].
j LexRank: LexRank is a graph based extractive summarization approach that uses the cosine similarity matrix to determine similarity between sentences and uses eigenvector centrality to extract relevant sentences [37]. A network of sentences is generated where each sentence corresponds to a node, and the edges represent the cosine similarity between pairs of sentences. The LexRank algorithm implemented in the MEAD toolkit was used for evaluation [38].

### Indicative summarization

A word-cloud based visualization method is used to represent term/concept distribution. This provides a concept-oriented summarization of the over-represented relevant terms and concepts in the input text. A weighted scoring scheme is used to prioritize terms corresponding to diseases/disorders, genes, mutations, and chemical names.

2 $$Score\left( t \right) = i*\left( {{\text{Wc}}} \right)*tf*IDF,$$

where *i* = 1 if the term is found in the controlled vocabulary, 0 otherwise, W_C_ = 1000 if the term is a disease/disorder, chemical, mutation, gene; 1 otherwise, tf = frequency of term t in the input text, IDF = $1 + {\text{log}}\left( {\frac{{\left( {total\,number\,of\,indexed\,Medline\,abstracts} \right)}}{{\left( {number\,of \,abstracts\,with\,term\,t} \right)}}} \right)$.

The weight, ${\text{Wc}},$ is selected to reflect the user-chosen term emphasis on certain clinical and disease characteristics for the summarization. Other values can be used depending on the type of summarization purpose and emphasis. IDF, the inverse document frequency, measures how common/rare a term is in the corpus.

### Interactiive user-guided summarization

Visual data exploration provides insights into the data and makes the data mining process more effective by incorporating human perception and intelligence [26]. CERC facilitates visual mining by means of an interactive word cloud. The word cloud represents the distribution of the relevant terms in the input text and can be used to interactively filter the ranked list of sentences to generate keyword-based summaries. Alternatively, users can manually define the keywords for filtering the ranked sentences to generate query-specific summaries.

### Evaluation

The CRAFTtest corpus (consists of 32 full text articles from CRAFT) and a set of 30 randomly selected full-text clinical case reports from BMC Ophthalmology, BMC Neurology, BMC Pulmonary Medicine, BMC Cancer, and New England Journal of Medicine were used to measure the performance of the three sentence ranking methods: MINTS, LexRank, and topicDist. For the clinical case-reports, the criteria for inclusion included availability of both abstract and full-text from journals focusing on different clinical conditions. No other annotations were available for the clinical case reports. Position-based ranking and random selection were used as baseline. In the position-based selection, sentences were assigned scores according to their position in the document with the earlier the higher. An extractive summary was generated using each method from the full text of the articles using the top five sentences. The summaries generated by each method were compared with the human generated summaries (abstracts) using Recall-Oriented Understudy for Gisting Evaluation (ROUGE), a software package for evaluating and comparing summaries based on the n-gram co-occurrence statistics. using a recall based approach [39]. ROUGE-1 and ROUGE-2 evaluate the overlap of unigrams and bigrams between the system generated and reference summaries, while ROUGE-SU4 evaluates the bigrams and allows a maximum skip distance of 4 between bigrams.

A one-sided paired Wilcoxon signed-rank test was used to evaluate the significance of differences between the ROUGE scores for randomly generated summaries and different summarization algorithms. The average performance of three randomly generated summaries was used for comparison.

## Results

### Content summarization

**Fig. 3:**
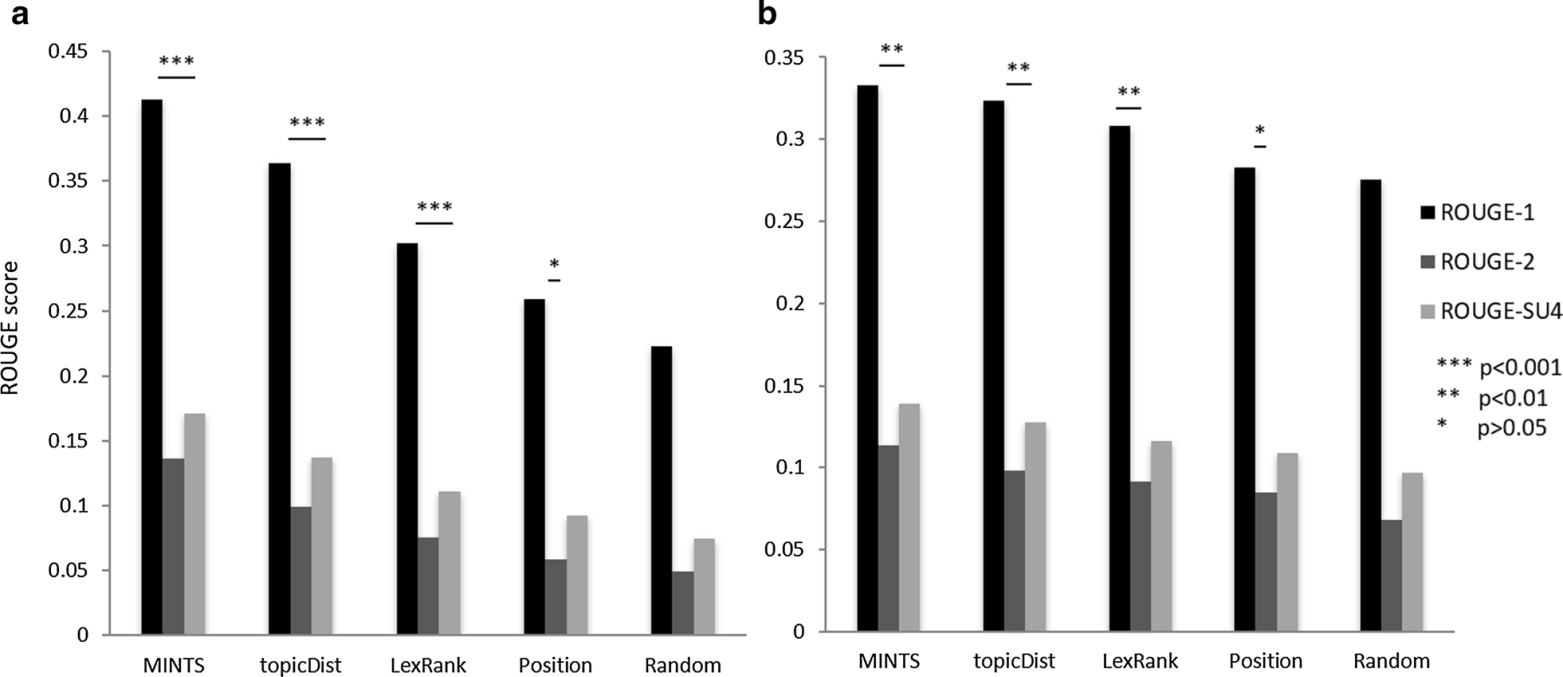
ROUGE evaluation scores for different extractive summarization methods using abstracts as gold standard summaries. **a** Performance comparison of different summarization approaches on CRAFTtest corpus (32 full-text scientific articles); **b** Performance comparison of different summarization approaches using ROUGE evaluation toolkit on clinical case reports corpus. The p-values for ROUGE-1, ROUGE-2, and ROUGE-SU4 scores for each method were compared against respective baseline scores using a one-sided Wilcox text. The MINTS algorithm outperformed other methods in both cases

The random forests model achieved an out-of-bag classification accuracy of 87.78% on the training set. An overall classification accuracy of 87.5% and a balanced error rate (group-specific accuracy) of 79.4% (94.6% for “good” category and 64.2% for “bad” category) was achieved for the blinded test set of 3,812 sentences. The ROUGE evaluation scores of extractive summaries generated using different methods are shown in Fig. 3. MINTS gave the best performance in both experiments. For the CRAFTtest corpus of 32 full-text articles, MINTS gave ROUGE-1, ROUGE-2, and ROUGE-SU4 scores of 0.414, 0.136, and 0.171, respectively, with p-values from the one-sided Wilcoxon signed rank test ranging from 10–13 to 10–8 for the three scores (Fig. 3a). MINTS performed the best with 15% and 38% improvement in ROUGE-1 scores when compared to the topicDist and LexRank, respectively. Both topicDist and LexRank methods performed better than the baseline.

Similar ranking pattern was observed for the different extraction methods using the clinical case reports corpus as evaluation set (Fig. 3b). However, the p-values were higher when compared to the CRAFTtest evaluation (0.001 to 0.01), which is likely due to the differences in the lengths of the documents in the two corpuses. The number of sentences in the clinical case reports corpus ranged from 18 to 72, while the number of sentences in the CRAFTtest corpus ranged from 101 to 455. As described in “Methods”, users have the option to specify the number of sentences to be used for generating the document summary.

### Advancing clinical translational research [41, 42]

#### An automated language translation system

We apply CERC within an automated language translation system for clinical usage [42]. Language barriers hinder communication and interaction between patients and clinicians. Yet, proper communication is critical for optimal patient care and best outcomes [41]. In the year 2014, Children’s Healthcare of Atlanta (CHOA) cared for approximately 27,000 patients (77,000 visits) with limited English proficiency (LEP). To improve patient-provider communication for patients with LEP, it is necessary to interpret spoken language and translate written clinical documents that need to be shared with the patients, to their primary language of communication. Currently, there is a gap in the standard of care with patients with LEP not getting the discharge summaries in the language they can comprehend. Mounting evidence has shown that LEP is a risk factor for reduced healthcare accessibility, reduced quality of care, decreased patient satisfaction, poor understanding of provider’s instructions, increased length of hospital stays, and increased adverse events and misdiagnoses.

People with LEP are also less likely to take advantage of preventive care such as immunizations, eye and dental care, cancer screening and other services [47–49]. Thus, limited patient–provider communication due to the language barriers can negatively impact and burden payers, providers and the community as a whole. It has been shown that utilization of professional language interpreter services by healthcare providers reduces the risks associated with poor communication due to language barriers [50]. The quality of care for patients with LEP can be improved with qualified interpreters, including reduction of communication errors and disparities of care, and improvement in clinical outcomes, and patient satisfaction [50–52]. Further, research indicates that it is cost-effective to provide interpreter translation services as it reduces unnecessary testing, shortens visit times, and improves compliance with treatment and follow-up instructions [53, 54].

Despite the above facts, professional language interpreters are under-used by providers, especially physicians with inadequate second language skills [51]. The providers often instead use family members, friends, and other staff or manage with their own limited language skills for interpretations during patient care [55, 56]. But, use of such ad-hoc interpreters has been linked to communication errors thus compromising privacy, quality and safety of healthcare services [57, 58].

As a pilot, we sought to integrate translation within the day-to-day care process of healthcare providers. We translated Emergency Department discharge summaries using computer-assisted translation and machine translation, from English to two of the most spoken other languages by the CHOA LEP population.

**Fig. 4:**
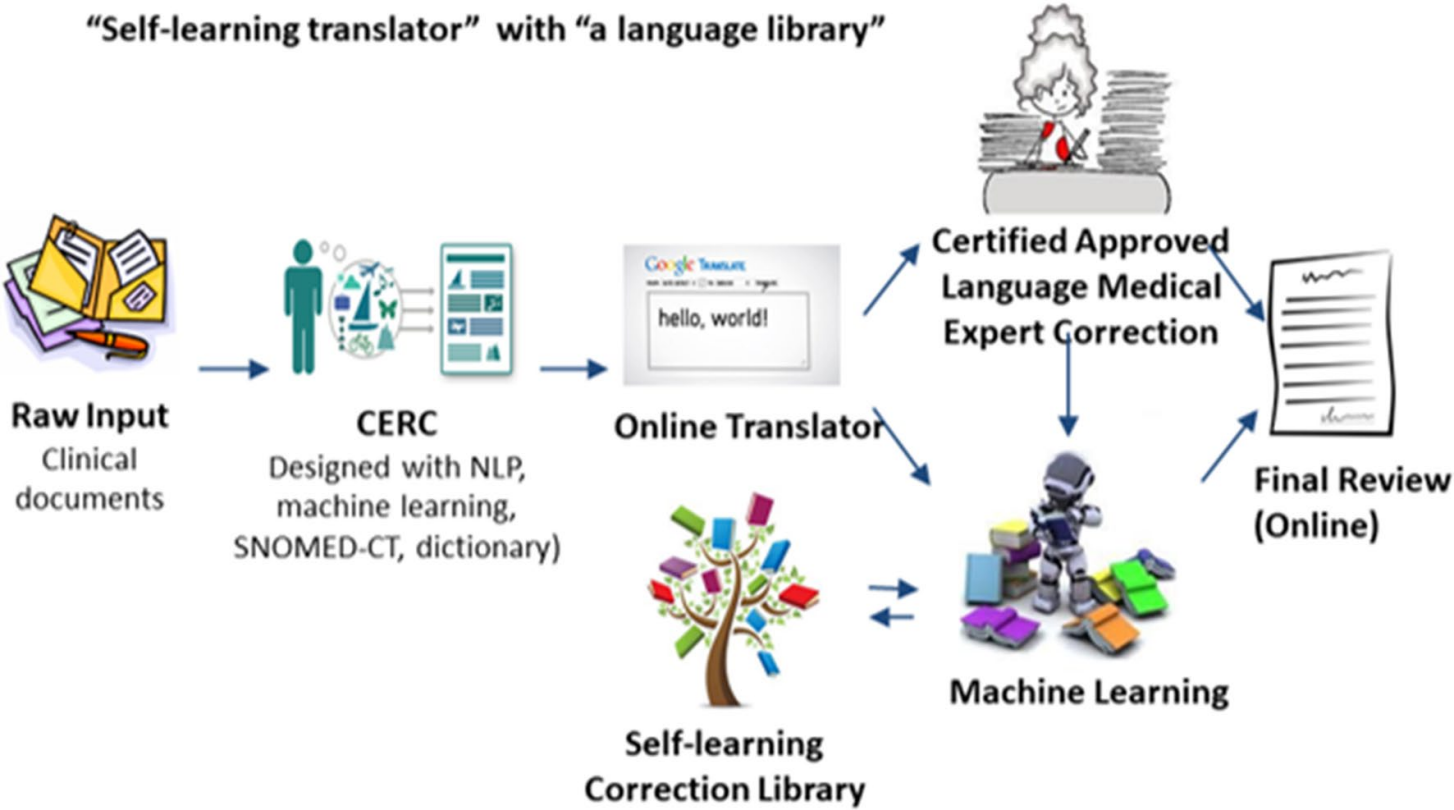
The automatic language translator design schema. The system utilizes a machine learning environment that incorporates CERC, Google Translate, a “self-learning translator,” and “a language library”. CERC first processes narrative text from de-identified discharge summaries, Google Translate then translates the resulting summary from English into different languages. Professional language experts correct the translated text and the self-learning translator takes in the processed discharge text as well as the expert corrected content, learns adaptively from the corrections and retains that knowledge in its self-learning library

We designed an automatic language translator that utilizes a machine learning environment that incorporates CERC, Google Translate, a “self-learning translator,” and “a language library” (Fig. 4). CERC first processes narrative text from de-identified discharge summaries, Google Translate then translates the resulting summary from English into different languages. Professional language experts correct the translated text and the self-learning translator takes in the processed discharge text as well as the expert corrected content, learns adaptively from the corrections and retains that knowledge in its self-learning library.

We evaluate the performance using the bilingual evaluation understudy (BLEU) algorithm [43]. Scores are calculated for individual translated segments (sentences) by comparing them with a set of good quality reference translations. This approximates the human judgement at a corpus level. The output BLEU value is between 0 and 1, with values closer to 1 indicating more similar (thus a better translation).

Table 1 shows that the performance of the language translator is significantly better for Spanish (0.864 vs 0.293) and Vietnamese (0.568 vs 0.199) using CERC when compared to without using CERC for initial summarization. This demonstrates CERC is a promising summarization tool, and that the training set can produce clinically acceptable results.

**Table 1:** shows that the performance of the language translator is significantly better for Spanish (0.864 vs 0.293) and Vietnamese (0.568 vs 0.199) using CERC when compared to without using CERC for initial summarization

	With CERC	Without CERC
Strep Throat Document (English- > Spanish)	0.864	0.293
Strep Throat Document (English- > Vietnamese)	0.568	0.199

The translator can be generalized across a broad range of clinical settings and patient populations where language barriers are of concern, demonstrating the clinical value of CERC for patient care. As a large corpus is fed, both the self-learning translator and the language library will expand their vocabulary and related content. We will continue to refine CERC using a larger training set which can lead to better sentence evaluation and summarization results.

### Facilitating clinical decision making

The system has several additional features to enhance clinical decision-making:

i Document-driven search to retrieve related literature from Medline: CERC uses the clinically/biologically relevant terms to find related articles in PubMed. This allows users to gain additional information about the key diseases or medications that are mentioned in the input text.
j Visualization of over-represented terms using controlled dictionary (PubTator, MeSH and SNOMED CT): The system uses the term-frequency criteria to identify clinically/biologically relevant terms in the input text. A word cloud representation of the top clinically/biologically relevant terms is generated. This could facilitate detection of high-risk findings
k Interactive interface and visualization: The web interface allows users to generate and edit automated summaries from the ranked sentences. Users have the option to filter sentences by keywords and generate a summary of the document based on the relevant sentences.
l Library of summaries: The system allows the users to automatically generate, edit, and save summaries for downstream pattern mining.

**Fig. 5:**
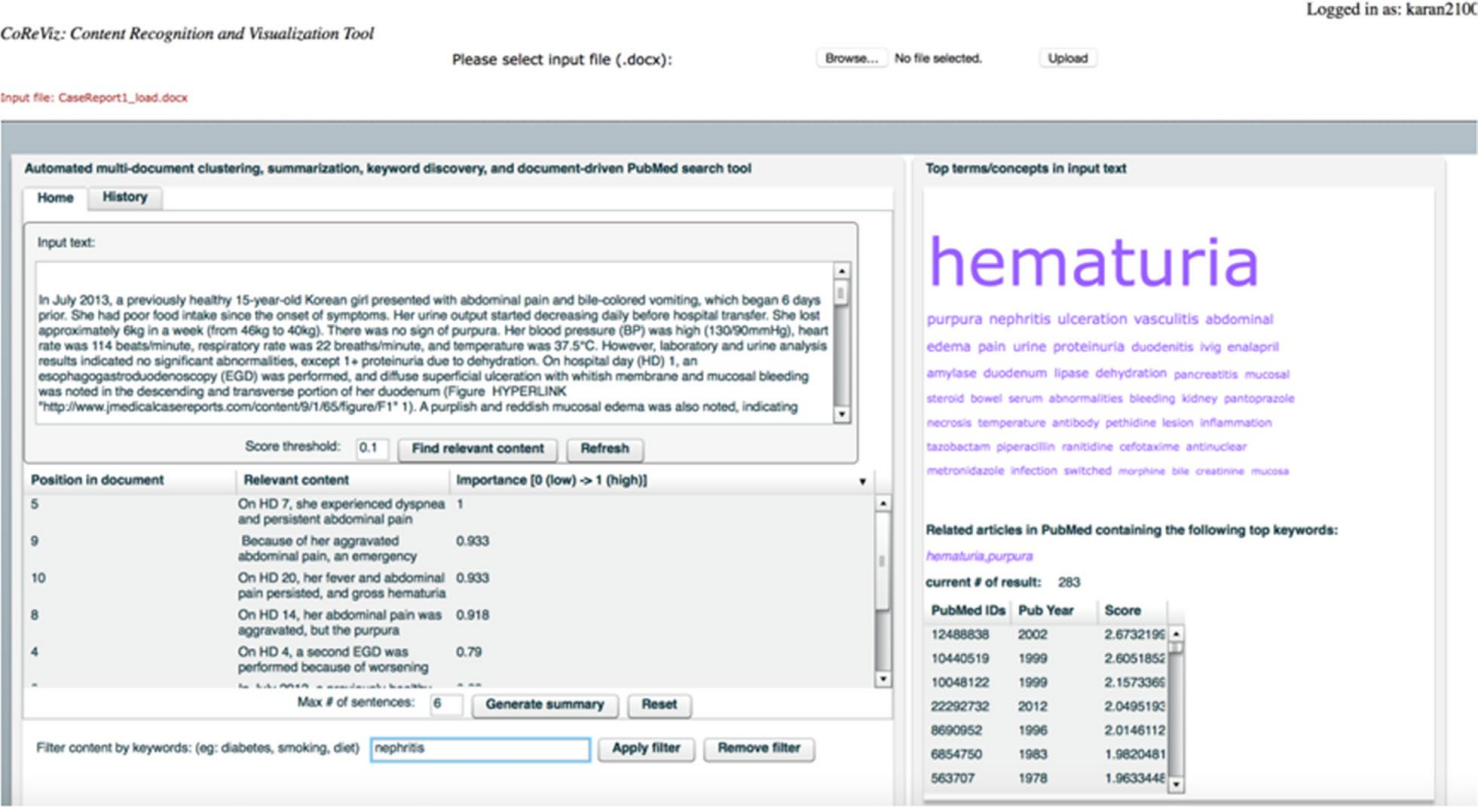
CERC demonstration. Users can upload or paste the input document and select the clustering and summarization options. The output includes a word cloud of over-represented clinical/biomedical terms, ranked sentences within each cluster, and related articles in Medline. Users can filter the list of ranked sentences based on keywords

Figure 5 shows an illustration of the system. Users can use the copy/paste option or upload a Word document with input text. A table with relevance scores for each sentence is returned based on the newly developed MINTS algorithm. Users can filter the sentences based on keywords, e.g. “diabetes”. Alternatively, the interactive word cloud can be used for filtering the sentences by clicking on the term of interest. An extractive summary can be generated using the top *N* sentences, where *N* is a user-defined parameter.

Users have the option to edit and save the generated summary for future analysis such as temporal tracking of clinically relevant indicators or medication usage. The system also provides a list of related PubMed articles based on the top over-represented terms in the input text.

#### A customizable information extraction and pre-processing pipeline for EHR

We demonstrate the use of CERC within a customizable information extraction and pre-processing pipeline for EHRs which extracts, anonymizes, and encrypts data directly from EHRs [63, 64]. Specifically, CERC is used for information extraction from extracted narrative clinical texts. Below is a short excerpt from our paper [64].

Prostate cancer is the most frequently diagnosed cancer in 105 countries and the fifth leading cause of cancer death in men [59]. The American Cancer Society estimated that there will be 191,930 new cases of prostate cancer in the U.S. in 2020 with an associated 33,330 deaths. Early prostate cancer detection has been achieved through prostate-specific antigen (PSA) test and biopsy of tissue removed during prostatectomy or at autopsy [59]. Through mathematical modelling [60], concluded that under the assumption that stage shift implies survival shift–which motivates early detection of cancer, PSA screening likely explains half or more of the mortality reduction observed in the U.S. since the early 1990s. EHR provides long-term tracking of patient PSA test results. These longitudinal data can be extracted using the lab component IDs or names of the test procedure. The rate of increase in PSA level, often represented using PSA doubling time or PSA velocity, has been widely used in the management of prostate cancer [61, 62].

#### Information extraction from EPIC EHR database

The extracted dataset covers 98,806 patients with the ICD-9 code 790.93 or ICD-10 code 97.20, “elevated prostate specific antigen (PSA)”. This dataset spans the years 1997–2018 and is composed of patient-level data (70 Mb), problem lists (384 Mb), medications (7.3 Gb), billing (167 Mb), laboratory orders (10 Gb), and clinical notes (46.1 Gb), totaling 64.02 Gigabytes. Patient IDs were successfully encrypted using SHA-256 encryption. PHI including patient names, addresses, institutions, age, phone numbers, and email addresses were detected and encrypted into dummy tokens.

We applied CERC and clinical concept extraction system on a subset of patients treated with radioactive seed implants. An additional 2,194 standardized clinical features were extracted from their clinical notes, including “Chronic pain syndrome”, “Placement of stent”, “Nerve conduction testing”, “Vascular Calcification”, “Overweight”, “Obstructive sleep apnea syndrome”, “Neoplasm, metastatic”, and “Lithotripsy”, etc.

Patient PSA laboratory test results were used as indicators of disease severity. PSA records were retrieved by the following method: (1) component IDs for lab records matching the query string “%PSA%” were retrieved; (2) PSA-irrelevant lab components were discarded, leaving 10 unique component IDs corresponding to “PSA-screening”, “PSA-monitoring”, “PSA”, “PSA FREE”, “PSA % FREE”, “PSA, external result”, “PSA, MHS”, “PSA with reflex FPSA, external result”, “PSA, screening”, and “PSA, cancer monitoring”; (3) “PSA FREE” and “PSA % FREE” were removed from the list of candidate components since free PSA is reported as a percentage of the total that is not protein bound, i.e., free. The higher the free PSA, the lower the likelihood of cancer; (4) PSA lab records were then retrieved by patient IDs and the filtered component IDs; (5) Missing, erroneous, and duplicated records were removed, and the remaining records were sorted by date and transformed into time series format for each patient.

#### Data standardization to SNOMED-CT Using SNOMED-CT

ontology as the mapping standard, we successfully mapped 22,842 out of the 39,570 unique clinical concepts. These 22,842 concepts were mapped to 4,673 unique SNOMED- CT concepts. Table 2 shows the number of unique concepts before mapping, with available mapping, and the number of SNOMED-CT concepts mapped to. Through this process, we significantly reduced the feature dimension, removed data redundancy and inconsistency, and lowered the likelihood of data collinearity. This establishes an interoperable cohort of patients. Users can apply clustering and machine learning for evidence-based treatment planning discovery or other comparative effectiveness and personalized treatment advances [64].

**Table 2:** Mapping results for labs, medications, and procedures data using the extracted content from CERC

	Lab	Procedure	Medication
Total unique concepts (39,570)	3662	2760	33,148
Number of unique concepts with direct mapping	1267	696	952
Number of unique concepts with indirect mapping	1588	1284	17,055
Number of unique SNOMED-CT concepts mapped to	1100	1170	2403

## Discussion

The success of new healthcare initiatives such as the Precision Medicine Initiative relies critically on the ability of computational tools and algorithms to address challenges related to efficient and impactful usage of information existing in different data sources. The vast amount of information in electronic health records and scientific literature has the potential to enhance clinical decision-making and improve the quality of healthcare as more informed decisions can be made at the patient level by integrating knowledge in the biomedical domain with patient characteristics and medical history [2, 7, 8]. However, the growing sizes of biomedical and clinical databases have created the problem of “information overload” [3]. A large amount of information in the healthcare domain such as clinical notes, discharge summaries, radiology reports etc. is stored in the form of text. Most existing text summarization tools for clinical/biomedical domain utilize single indicators of relevance such as concept distribution, position, and rely upon UMLS as the main vocabulary for identifying concepts and semantic relations between concepts, which limits the incorporation of specialized biomedical terminology such as genetic variants [5]. In addition to natural language processing, visualization techniques are essential for representation of information in a form that facilitates pattern recognition and large volumes of data [27, 28].

We developed a web-based content recognition and summarization tool, CERC (https://newton.isye.gatech.edu/CERC1/), for clinical and biomedical text that includes features such as extractive summarization to identify relevant sentences, indicative summarization of the overrepresented biomedical terms and concepts in the input text using word cloud visualization, interactive concept-oriented summarization, and retrieval of biomedical literature relevant to the input text (Fig. 4). A controlled vocabulary dictionary generated using MeSH, SNOMED-CT, and PubTator is used for determining relevant terms.

Extractive summarization is performed using a new algorithm, the MINTS algorithm. MINTS uses a multi-stage framework that combines supervised learning techniques, individual characteristics of sentences (position, length, relevant terms) and network level characteristics (degree centrality) for extracting salient sentences. A random forests classifier trained on a set of 9779 sentences from 35 full-text articles from the CRAFT corpus is used for evaluating sentence worthiness for summarization, “good” vs “bad”. Multiple indicators of importance such as degree centrality, presence and number of relevant terms, and position are used during relevance evaluation and ranking stages. An aggregated ranking scheme and cosine similarity-based redundancy evaluation is used for selecting top sentences. Redundancy detection is performed using cosine similarity between potential candidates and already selected sentences.

The performance evaluation results on full-text scientific articles and clinical case reports demonstrate improved summarization process that is achieved by combining machine learning, text mining, network analysis techniques with domain knowledge as opposed to using single characteristics of relevance [16, 17]. Furthermore, the results suggest that the length of the input text does not affect the performance of the MINTS algorithm. The two corpuses varied in their sizes as well as structure and content as the clinical case reports focus on diagnosis, treatment, and management of clinical cases and are targeted towards clinical audience, while the scientific articles focus on basic science or biomedical research. These results demonstrate the promise of “intelligent” algorithms like MINTS in addressing the issue of information overload in both the clinical and biomedical domains.The automatic language translator and the customizable information extraction and pre-processing pipeline for EHR demonstrate that CERC can readily be incorporated within clinical decision support systems to improve quality of care and to assist in data-driven and evidence-based informed decision making for direct patient care.

### Limitations

First, although the evaluation was performed on different types of full-text articles from both biomedical and clinical domain, further validation is required including extrinsic assessment by clinicians. Second, the terms in the topic cloud are currently not mapped to their corresponding concepts leading to ambiguity and redundancy if a concept is represented in different forms in the input text. Third, the random forests classifier was built using only a subset of all possible indicators of relevance leaving room for improvement at the initial sentence evaluation level [17]. Furthermore, the classifier was built using an imbalanced dataset which led to a low balanced accuracy of 79.4% and a lower accuracy for the “bad” group (64.2%) compared to the “good” group (94.6%). Evaluation of performance of different classification algorithms [63] or using a larger training set can lead to better sentence evaluation and summarization results. Fourth, the algorithms used for indicative and extractive summarization do not utilize lexical or semantic relationships between terms/concepts. A more detailed natural language analysis could further improve the performance of the summarization algorithms. Finally, the system currently supports only English language.

### Future work

Extrinsic evaluation of the system and further validation of the summarization strategies using different types of clinical text such as operative notes and radiology reports will be performed in a patient care setting. The evaluation will focus on the ability of the system for high-risk findings in patient records and the impact on patient care and clinical decision-making. The functionality of the system will be further extended by providing automated graph-based summarization of the input text as demonstrated in our previous work, SEACOIN, which was designed for topic-based summarization of Medline abstracts [40, 44]. The terms in the interactive cloud will be mapped to concepts in PubTator and SNOMED-CT [33, 34].

## Conclusion

Intelligent tools and techniques are required to extract information from rapidly growing data in healthcare and biomedical domain to facilitate precision medicine. In this work, we have developed CERC (https://newton.isye.gatech.edu/CERC1/), an interactive content recognition and summarization tool for extracting salient information from clinical and biomedical text. The system includes both indicative and informative summarization strategies that allow the users to retrieve and visualize important content from the input text in an interactive manner. A novel multi-stage procedure, MINTS, is introduced. The algorithm uses a random forests classifier to evaluate the “worthiness” of individual sentences for summarization prior to scoring based on multiple domain specific, sentence-level, and network-level characteristics. The ROUGE evaluation results on two independent test corpuses show that MINTS provides better summarization results when compared to methods based on single indicators (topic/concept frequency distribution and LexRank). ROUGE evaluation scores for the MINTS algorithm were significantly different when compared to random selection at a significance level of 0.01: ROUGE-1 (0.41 vs 0.22), ROUGE-2 (0.14 vs 0.06), and ROUGE-SU4 (0.17 vs 0.07) on CRAFTtest; and ROUGE-1 (0.33 vs 0.28), ROUGE-2 (0.11 vs 0.07), and ROUGE-SU4 (0.14 vs 0.1). The word cloud visualization provides a concept-oriented summary of the text and allows users to retrieve salient content according to their specific interests and requirements. The system can be used for summarizing and identifying relevant content from full-text articles from a variety of information sources such as Medline, Cochrane, UpToDate (http://www.uptodate.com/), and from clinical text such as clinical notes, radiology reports, etc. The system incorporates several features to address the challenges related to extracting information from large volumes of text. The automatic language translator and the customizable information extraction and pre-processing pipeline for EHR demonstrate that CERC can readily be incorporated within clinical decision support systems to improve quality of care and to assist in data-driven and evidence-based informed decision making for direct patient care. Future work will focus on extrinsic evaluation of the system in both patient care and research settings.

## Data Availability

The datasets used in this study involves SNOMED-CT, PubMed, and the CRAFT corpus. CRAFT: http://bionlp-corpora.sourceforge.net/CRAFT/. PubTator: ftp://ftp.ncbi.nlm.nih.gov/pub/lu/PubTatorCentral. MeSH: https://www.ncbi.nlm.nih.gov/mesh. SNOMED-CT: http://www.snomed.org/. PubMed Case reports: 1. https://pubmed.ncbi.nlm.nih.gov/23570263/ 2. https://pubmed.ncbi.nlm.nih.gov/23617826/ 3. https://pubmed.ncbi.nlm.nih.gov/25967676/ 4. https://pubmed.ncbi.nlm.nih.gov/26001650/ 5. https://pubmed.ncbi.nlm.nih.gov/26036321/ 6. https://pubmed.ncbi.nlm.nih.gov/25887242/ 7. https://pubmed.ncbi.nlm.nih.gov/25879889/ 8. https://pubmed.ncbi.nlm.nih.gov/25420956/ 9. https://pubmed.ncbi.nlm.nih.gov/25344209/ 10. https://pubmed.ncbi.nlm.nih.gov/24885608/ 11. https://pubmed.ncbi.nlm.nih.gov/25885466/ 12. https://pubmed.ncbi.nlm.nih.gov/25884435/ 13. https://pubmed.ncbi.nlm.nih.gov/25885098/ 14. https://pubmed.ncbi.nlm.nih.gov/25884640/ 15. https://pubmed.ncbi.nlm.nih.gov/25880568/ 16. https://pubmed.ncbi.nlm.nih.gov/19641205/ 17. https://pubmed.ncbi.nlm.nih.gov/19439744/ 18. https://pubmed.ncbi.nlm.nih.gov/25337633/ 19. https://pubmed.ncbi.nlm.nih.gov/25390460/ 20. https://pubmed.ncbi.nlm.nih.gov/24195502/ 21. https://pubmed.ncbi.nlm.nih.gov/24716661/ 22. https://pubmed.ncbi.nlm.nih.gov/19741228/ 23. https://pubmed.ncbi.nlm.nih.gov/19246360/ 24. https://pubmed.ncbi.nlm.nih.gov/21345103/ 25. https://pubmed.ncbi.nlm.nih.gov/22931317/ 26. https://pubmed.ncbi.nlm.nih.gov/25924662/ 27. https://pubmed.ncbi.nlm.nih.gov/25887519/ 28. https://pubmed.ncbi.nlm.nih.gov/26082835/ 29. https://pubmed.ncbi.nlm.nih.gov/26066034/ 30. https://pubmed.ncbi.nlm.nih.gov/26071911/

## Abbreviations

BLEU: Bilingual Evaluation Understudy
CERC: A content extraction, recognition, and construction visualization tool
CRAFT: Colorado Richly Annotated Full Text
DS: Sørensen–Dice-coefficient/index
EHR: Electronic health record systems
IDF: The inverse document frequency
MINTS: Multi indicator text summarization
PSA: Prostate-specific antigen
ROUGE: Recall-Oriented Understudy for Gisting Evaluation
TF-IDF: Term frequency–inverse document frequency
